# The Next Target for Readmission Reporting? Exploring Readmission Rates of Patients with CLABSI

**DOI:** 10.1017/ash.2024.198

**Published:** 2024-09-16

**Authors:** Caitlin Crews-Stowe, Elliot Sklar

**Affiliations:** University of Tennessee at Chattanooga; Nova Southeastern University

## Abstract

**Background:** Multi-drug resistant organisms (MDROs) are a common cause of healthcare-associated infections, particularly central line-associated bloodstream infections (CLABSIs). Prior research has shown that MDROs cause up to 67% of CLABSIs and have up to a 37% increase in 30 day readmission, which is higher than readmission rates for other conditions reported to the Centers for Medicare and Medicaid Services (CMS). The objective of the study was to determine overall 90-day readmission rates, and if there was a difference in readmission rate within 90 days post discharge for patients who had a MDRO as the causative pathogen of their CLABSI compared to patients who did not have an MDRO. **Methods:** A retrospective analysis of patient data from a nine-hospital system was performed on patients who had a CLABSI and were discharged alive between January 1st, 2018, and December 31st, 2019. Basic descriptive statistics were performed, and the potential differences in readmission rates were examined using Chi-square analyses. **Results:** The overall readmission rate for all CLABSIs was 46.9%. The chi-square analysis determined there was not a significant difference in readmission rates in patients who had a MDRO CLABSI compared to patients with a non-MDRO CLABSI (59.1% vs. 44.6%, x2= 1.564 , p= 0.211). **Conclusion:** There was not a significant difference in readmission rates between patients with an MDRO CLABSI compared to a non-MDRO CLABSI. However, the overall readmission rate for this patient population was much higher than seen in previous literature and other publicly reported readmission rates. Additional research is recommended to explore if the increased CLABSI readmission rates seen are a unique finding to this health system.

